# NT-proBNP, echocardiographic abnormalities and subclinical coronary artery disease in high risk type 2 diabetic patients

**DOI:** 10.1186/1475-2840-11-19

**Published:** 2012-03-05

**Authors:** Henrik Reinhard, Peter R Hansen, Niels Wiinberg, Andreas Kjær, Claus L Petersen, Kaj Winther, Hans-Henrik Parving, Peter Rossing, Peter K Jacobsen

**Affiliations:** 1 Steno Diabetes Center, Niels Steensenvej 1, DK-2820 Gentofte, Denmark; 2 Department of Cardiology, Gentofte University Hospital, Gentofte, Denmark; 3 Department of Clinical Physiology and Nuclear Medicine, Frederiksberg University Hospital, Frederiksberg, Denmark; 4 Department of Clinical Physiology, Nuclear Medicine & PET and Cluster for Molecular Imaging, Rigshospitalet, University of Copenhagen, Copenhagen, Denmark; 5 Department of Clinical Biochemistry, Frederiksberg University Hospital, Frederiksberg, Denmark; 6 Department of Medical Endocrinology, University Hospital of Copenhagen, Copenhagen, Denmark; 7 Faculty of Health Science, Aarhus University, Aarhus, Denmark; 8 The Heart Centre, Rigshospitalet, Copenhagen, Denmark

## Abstract

**Background:**

Intensive multifactorial treatment aimed at prevention of cardiovascular (CV) disease may reduce left ventricular (LV) echocardiographic abnormalities in diabetic subjects. Plasma N-terminal (NT)-proBNP predicts CV mortality in diabetic patients but the association between P-NT-proBNP and the putative residual abnormalities in such patients are not well described. This study examined echocardiographic measurements of LV hypertrophy, atrial dilatation and LV dysfunction and their relation to P-NT-proBNP levels or subclinical coronary artery disease (CAD) in type 2 diabetic patients with microalbuminuria receiving intensive multifactorial treatment.

**Methods:**

Echocardiography including tissue Doppler imaging and P-NT-proBNP measurements were performed in 200 patients without prior CAD. Patients with P-NT-proBNP > 45.2 ng/L and/or coronary calcium score ≥ 400 were stratified as high risk patients for CAD(n = 133) and examined for significant CAD by myocardial perfusion imaging and/or CT-angiography and/or coronary angiography.

**Results:**

LV mass index was 41.2 ± 10.9 g/m^2.7 ^and 48 (24%) patients had LV hypertrophy. LA and RA dilatation were found in 54(27%) and 45(23%) patients, respectively, and LV diastolic dysfunction was found in 109(55%) patients. Patients with increased P-NT-proBNP levels did not have more major echocardiographic abnormalities. In 70(53%) of 133 high risk patients significant CAD was demonstrated and patients with LV hypertrophy had increased risk of significant CAD(adjusted odd ratio[CI] was 4.53[1.14-18.06]).

**Conclusion:**

Among asymptomatic type 2 diabetic patients with microalbuminuria that received intensive multifactorial treatment, P-NT-proBNP levels is not associated with echocardiographic abnormalities. LV diastolic dysfunction was frequently observed, whereas LV hypertrophy was less frequent but associated with significant CAD.

## Introduction

Current multifactorial treatment strategies for cardiovascular (CV) disease aimed at reduction of established conventional risk factors are effective [1], but not sufficient to overcome the increased CV mortality in diabetic patients [2]. We have previously identified elevated plasma N-terminal-pro-brain natriuretic peptide (P-NT-proBNP) as a strong predictor of CV mortality in type 2 diabetic patients [3]. The causes of poor prognosis associated with elevated P-NT-proBNP levels are not known in detail but P-NT-proBNP levels correlate with echocardiography measurements in diabetic populations not receiving multifactorial treatment [4]. Echocardiography in asymptomatic high CV risk type 2 diabetic patients may disclose heart abnormalities [5]. Selected echocardiographic abnormalities, including left ventricular (LV) systolic and diastolic dysfunction are related to CV risk factors and adverse CV events [5,6]. For example, the prevalence of LV hypertrophy was high (43%) in asymptomatic type 2 diabetic patients without CV disease and not taking antihypertensive medications [7], and LV diastolic dysfunction was present in almost 50% of asymptomatic subjects with newly diagnosed type 2 diabetes [4].

Over the last decades, however, the intensive multifactorial treatment targeting hyperglycaemia, hypertension, dyslipidaemia and life style factors to achieve CV protection may have reduced the previously reported high prevalence of LV hypertrophy and LV dysfunction in diabetic subjects. Indeed, we have previously reported that the prevalence of increased carotid intima-media thickness (CIMT > 1.0 mm) was surprisingly low in a cohort of 200 type 2 diabetic patients with microalbuminuria that received intensive multifactorial treatment [8]. LV hypertrophy may regress and LV contractility improve after treatment with drugs, e.g., renin angiotensin aldosterone system (RAAS)-blocking agents, that are now part of recommended multifactorial treatment [9]. This improvement is associated with lower CV mortality, but the magnitude of LV hypertrophy regression and CV risk reduction is less in diabetic patients compared to nondiabetic controls [9]. In addition, a 2002 UK study found that 44% of diabetic patients taking angiotensin-converting enzyme (ACE) inhibitors still had LV hypertrophy, independent of blood pressures [5]. Furthermore, the previously reported high prevalence of LV diastolic dysfunction in diabetic subjects has also been suggested to be resistant to medical treatment [5]. The extent of residual echocardiographic abnormalities and association with P-NT-proBNP levels has not been reported in asymptomatic diabetic patients with microalbuminuria that receive intensive multifactorial treatment. Furthermore, the well-described association between echocardiographic abnormalities, CV risk factors, and coronary artery disease (CAD) might be diluted in patients receiving multifactorial treatment with its attendant improved metabolic, lipid and blood pressure control [10]. Accordingly, this study examined echocardiographic abnormalities and their relation to P-NT-proBNP levels or subclinical significant CAD in type 2 diabetic patients with microalbuminuria that received intensive multifactorial treatment.

## Methods

### Patient cohort and investigations

In a cross-sectional study, we identified from January 2007 to February 2008, a consecutive cohort of 200 type 2 diabetic patients with microalbuminuria but without prior CAD or other cardiac disease and normal plasma creatinine (to allow for examinations with x-ray contrast media). All patients received intensive multifactorial intervention aimed at optimal glycaemic, lipid, and blood pressure control, as well as antiplatelet therapy and lifestyle modification according to the Steno-2 study [1]. The design, methods and cardiac examinations performed in this cohort have previously been described [10]. In brief, P-NT-proBNP was measured in all patients and analyzed with an immunoassay. Cardiovascular examinations in addition to echocardiography included CIMT measured at the posterior wall 20 mm proximal to the bifurcation bilaterally with a Siemens Acuson Cypress MHz ultrasound scanner and with calculation of the mean of measurements on both sides. We also calculated the carotid compliance (l/mmHg) as previously described [11]. Furthermore, systolic blood pressures in the ankle and big toe were measured on both legs by strain gauge technique and the lowers ankle and big toe pressures were recorded and calculated as ankle-brachial and toe-brachial blood pressure index [12]. Finally, the Agatston coronary calcium score (CCS) was measured using a 16 multidetector-row CT scanner (Philips Precedence MX 8000 IDT 16 slice, Philips Medical Systems, Best, The Netherlands) [13].

### Risk stratification and assessment of CAD

Patients were stratified into high risk and low risk groups based on elevated P-NT-proBNP and elevated CCS as follows [10]: 1) P-NT-proBNP > 45.2 ng/l or CCS ≥ 400 = high risk patients (n = 133); this P-NT-proBNP cut-off value was selected as the median value among the first 50 patients examined in the study and it corresponded well with our earlier findings [3], and 2) P-NT-proBNP ≤ 45.2 ng/l and CCS < 400 = low risk patients (n = 67). High risk patients were examined according to the following algorithm: 1) Patients with P-NT-proBNP > 45.2 ng/l underwent myocardial perfusion imaging (MPI);patients with abnormal MPI (n = 55) or CCS > 100 (n = 29) were referred for coronary angiography (CAG), 2) Patients with P-NT-proBNP ≤ 45.2 ng/l and CCS 400-1000 underwent CT angiography (CTA) (n = 20); CTA was only used in patients with CCS 400-1000 since heavy coronary artery calcifications (CCS > 1000) compromise the validity of CTA [14] and patients with abnormal CTA were referred for CAG (n = 15), and 3) Patients with P-NT-proBNP ≤ 45.2 ng/l and CCS > 1000 underwent MPI (n = 9); patients with abnormal MPI (n = 6) were referred for CAG. Significant CAD was defined as the presence of significant (reversible and/or irreversible) myocardial perfusion defects on MPI, and/or ≥ 70% stenosis of one or more major ( ≥ 2 mm) epicardial coronary arteries at CAG. The correlations between P-NT-proBNP, CTA, MPI and CAG have been reported previously [10].

### Echocardiography

Transthoracic echocardiography was performed using a Philips IE 33 machine (Phillips Medical Systems, Best, The Netherlands). All patients were examined with conventional 2-D and Doppler modalities, combined with colour tissue Doppler imaging. Data were stored on compact discs and analyzed off-line with commercially available software (EchoPAC software) by an experienced investigator, with no other relation to the present study and blinded to all other information.

### LV function

Simpson's apical biplane method was used to evaluate LV ejection fraction (LVEF) and left ventricular systolic dysfunction was defined as LVEF < 50% [15,16]. Pulsed wave Doppler examination of mitral inflow was performed at the tip of the mitral leaflets in the apical longitudinal chamber view. Peak velocities of early (E [m/s]) and atrial (A [m/s]) diastolic filling, deceleration time of the E wave (DT [ms]) and isovolumetric relaxation time (IVRT [ms]) were measured [15]. We also used tissue Doppler imaging of the LV basal annular longitudinal myocardial shortening as defined by the annular peak systolic velocity (s' [cm/s]) and the two lengthening velocities as defined by peak early mitral annular diastolic velocity (e'lateral [cm/s]) and peak atrial mitral annular diastolic velocity (a' [cm/s]), respectively. All tissue Doppler measurements were performed at the lateral border of the mitral annulus.

Normal LV diastolic function was defined as E/A ratio 0.7-1.5, DT 140-240 ms and e'lateral ≥ 10 [16,17]. Impaired LV relaxation (grade I diastolic dysfunction) was detected when the E/A ratio was < 0.7, DT > 240 ms and e'lateral < 10. A pseudonormal relaxation pattern (grade II diastolic dysfunction) was diagnosed when the E/A ratio was 0.7-1.5 ms, DT 140-240 ms and e'lateral < 10. Restrictive physiology (grade III diastolic dysfunction) was deduced when E/A ratio was > 1.5, DT < 140 and e'lateral < 10 [16,17]. Patients were required to display the specific Doppler criteria described above in order to be classified in a given category (grade) of LV diastolic dysfunction. Patients with only 1 or 2 criteria present or incomplete measurements were classified as having indeterminate diastolic dysfunction [18].

### LV structure

We calculated LV mass = (0.8 [1.04 (LVIDd + PWTd + SWTd)^3 ^-(LVIDd)^3^]) +0.6 g [15,19]. LV mass was indexed according to both body surface area (BSA = weight (kg)^0,425 ^× height (cm)^0,725 ^× 71,84 × 10^-4^) and body height in order to allow for comparison of data with other studies, and, importantly, we indexed LV mass according to height ^2.7 ^(LV mass/height^2.7^) because the latter index has been suggested to be optimal for indexing LV mass and grading of LV hypertrophy in obese subjects [15,20]. Normal values of LV mass and geometry according to recommendations [15]. In particular, LV hypertrophy according to LV mass/height^2.7 ^criteria was defined by LV mass index > 48 g/m^2.7 ^in men and > 44 g/m^2.7 ^in women, including moderate-severe LV mass as defined by LV mass index > 55 g/m^2.7 ^in men and > 51 g/m^2.7 ^in women, respectively. We also classified LV geometry in four mutually exclusive groups on the basis of LV mass and relative wall thickness (2 × PWTd)/LVDd) including 1) concentric hypertrophy (increased LV mass > 115 g/m^2 ^in men and > 95 g/m^2 ^in women and increased relative wall thickness > 0.42), 2) eccentric hypertrophy (increased LV mass and normal relative wall thickness), 3) concentric remodeling (normal LV mass and increased relative wall thickness), and 4) normal geometry (normal LV mass and normal relative wall thickness) [15].

Left atrial (LA) volume was measured in apical 4 chamber and 2 chamber views by the biplane area-length method and was indexed to BSA. Right atrial (RA) volume was measured in apical 4 chamber view by single area-length method and indexed to BSA. Mild, moderate and severe dilatation of LA or RA were defined as atrial volumes of 29-33, 34-39 and > 39 ml/m^2^, respectively, for both women and men [15]. Moderate-severe increase of LV diastolic volume was defined as LV diastolic volume/BSA > 86 ml/m^2 ^for both women and men [21].

The study was approved by the local ethics committee and all patients gave written informed consent.

### Statistical analysis

Our primary objective was to describe if patients with elevated NT-proBNP have more echocardiographic abnormalities. Accordingly, we investigated if patients with P-NT-proBNP levels above our previous defined cut-off value (45.2 ng/l) had more echocardiographic abnormalities than patients with P-NT-proBNP below this value [10]. Furthermore, we examined the associations between echocardiographic measurements and CV risk factors including P-NT-proBNP as a continuous variable in univariate and multivariate linear regression analyses. Finally, patients were divided into groups with or without significant CAD and here we assumed that the 67 low risk patients did not have significant CAD in agreement with their low P-NT-proBNP and CCS, and the comparatively good prognosis seen in these patients [10]. The association of echocardiographic variables with the presence of significant CAD were assessed by two logistic regression enter models and expressed as adjusted odds ratios (ORs) and 95% confidence intervals (CIs). All included CV risk factors and echocardiographic variables were selected according to significant associations observed in univariate analyses. The first logistic regression model included conventional echocardiographic data and the second model also included one tissue Doppler variable (E/e' lateral ratio). Due to co-linearity of different measurements of mitral inflow and tissue Doppler variables we only used E/A ratio and E/e' lateral ratio in these analyses, respectively. Finally, these analyses were also performed without inclusion of the low risk patients that did not undergo MPI, CTA and CAG examination. Data were expressed as means ± SD, except for non-normally distributed variables, which were log10-transformed before analysis and are given as medians (interquartile range). All data were analyzed by using statistical package for social sciences (SPSS) version 14 for Windows. A p value less than 0.05 was considered as statistically significant.

## Results

### Patient characteristics

The clinical characteristics of all patients and in patients with P-NT-proBNP levels below and above our cut-off value ( > 45.2 ng/l) are summarized in Table 1.

**Table 1:** The clinical characteristics of patients with plasma NT-proBNP below and above our defined cut-off value

	All patients (n = 200)	Patients with NT-proBNP ≤ 45.2 ng/l (n = 96)	Patients with NT-proBNP > 45.2 ng/l (n = 104)	p-values
Sex no. (male%)	76	78	74	0.51
Age (years)	59 ± 9	55.4 ± 10	61.7 ± 9	0.0001
Known duration of diabetes (years)	13 ± 7	10.8 ± 7	14.6 ± 7	0. 0.001
BMI (kg/m^2^)	32.6 ± 5.8	32.9 ± 5.6	32.3 ± 5.9	0.48
HbA_1c _(%)	7.9 ± 1.3	8.00 ± 1.3	7.7 ± 1.4	0.18
Systolic blood pressure (mmHg)	130 ± 17	131 ± 16	130 ± 18	0.55
Diastolic blood pressure (mmHg)	75 ± 11	76 ± 10	74 ± 12	0.18
Total cholesterol (mmol/l)	3.9 ± 0.9	4.1 ± 0.9	3.8 ± 1.0	0.032
Plasma creatinine (μmol/l)	76 ± 18	71.9 ± 16	80.6 ± 19	0.001
Haemoglobin (mmol/l)	8.6 ± 0.85	8.8 ± 0.9	8.4 ± 0.9	0.001
Heart rate variability during deep breathing (bpm) *	7 (4.5-11.5)	8 (42-205)	6 (4-10)	0.0001
Vibratory perception threshold mV - mean of both sides	33 ± 15	28.5 ± 14	37.9 ± 14	0.0001
Retinopathy no. (%)	120 (60)	44 (45)	76 (73)	0.0001
Urinary albumin excretion rate (mg/24 h)*	103 (39 - 230)	103 (38 - 491)	101 (38 - 286)	0.70
Peripheral systolic blood pressure (mmHg - worst big toe)	121 ± 34	125 ± 32	117 ± 35	0.35
Carotid intima-media thickness (mm)	0.73 (0.15)	0.71 ± 0.16	0.75 ± 0.14	0.15
Carotid compliance ^a ^(l/mmHg)	0.0026 ± 0.001	0.0025 ± 0.001	0.0026 ± 0.001	0.30
Coronary Calcium Score*	183 (0-4529)	87 (1-426)	251 (24-872)	0.014
Oral antidiabetic medication no. (%)	170 (85)	85 (89)	85 (82)	0.18
Insulin treatment no. (%)	124 (62)	59(61)	65 (63)	0.88
RAAS blockade no. (%)	196 (98)	94 (98)	102 (98)	0.94
Statin therapy no. (%)	189 (95)	91 (95)	98 (94)	0.82
Aspirin therapy no. (%)	183 (92)	87 (91)	96 (92)	0.80
Beta-blocker therapy no. (%)	27 (14)	4 (4)	23 (22)	0.0001
Calcium channel blockers no. (%)	80 (40)	36 (36)	44 (44)	0.25
Diuretics no. (%)	128 (64)	56 (58)	72 (69)	0.11

Our plasma NT-proBNP cut-off value has previously been defined but represented the median NT-proBNP in the first 50 patients examined in the study [10].

p-values reflect comparison between patients with high and low plasma NT-proBNP levels

Data are expressed as means (SD) or medians, interquartile range*

Of note, the multifactorial medical treatment for CV disease prevention included statins (94% of patients), aspirin (90%), and RAAS-blocking agents (98%). Furthermore, 33%, 28%, and 17% received 2, 3, and ≥ 4 antihypertensive drugs on top of RAAS blockade.

### Prevalences overall of echocardiography abnormalities

Echocardiography was completed in 99% (198/200) of the patients. One patient died from liver cancer before echocardiography and the data from another subject was deleted by accident. Significant valvular disease was seen in one patient with moderate aortic valve insufficiency, where LV size and systolic function were normal. Echocardiographic measurements are shown in Table 2.

**Table 2:** The echocardiographic measures of patients with plasma NT-proBNP below and above our defined cut-off value

	All patients (n = 200)	Patients with NT-proBNP ≤ 45.2 ng/l (n = 96)	Patients with NT-proBNP > 45.2 ng/l (n = 104)	p-values
Left ventricular structure				
Left ventricular end-diastolic volume [ml]	127.0 ± 28.9	129 ± 28.5	130 ± 31.9	0.85
Left ventricular end-systolic volume [ml]	52.0 ± 15.5	53.5 ± 16.7	53.9 ± 16.7	0.74
Left atrial volume index biplane [ml/m^2^]	25.6 ± 6.4	24.5 ± 6.1	26.7 ± .6.7	0.021
LV mass, g,	188.8 ± 52.6	203.1 ± 48.0	180.9 ± 53.5	0.11
LV mass/height^2,7^, [g/m^2.7^]	41.2 ± 10.9	39.85 ± 10.8	42.43 ± 10.7	0.10
Left ventricular systolic function				
Left ventricular ejection fraction [%]	59.5 ± 4.8	58.6 ± 5.7	59.9 ± 4.3	0.50
Peak mitral annular systolic velocity (s') [cm/s]	8.49 ± 1.58	8.71 ± 1.60	8.28 ± 1.54	0.057
Left ventricular diastolic function and grade				
Isovolumetric relaxation time [ms]	86.1 **± **15.8	88.3 **± **17.6	84.8 **± **14.7	0.14
Mitral valve E-wave deceleration time [ms]	201.0 **± **39.5	203.8 **± **38.0	199.6 **± **40.4	0.48
Mitral valve E-wave deceleration slope [m/s2]	3.7 **± **1.1	3.7 **± **1.1	3.8 **± **1.1	0.088
Peak mitral valve E-velocity [m/s]	0.71 ± 0.15	0.71 ± 0.15	0.71 ± 0.15	0.95
Peak mitral valve A-velocity [m/s]	0.93 ± 0.22	0.82 ± 0.17	0.77 ± 0.17	0.033
E/A ratio	0.93 ± 0.20	0.94 ± 20	0.92 ± 0.23	0.60
Peak early mitral annular diastolic velocity (e' lateral) [cm/s]	8.86 ± 2.34	9.23 ± 2.55	8.50 ± 2.06	0.032
Peak atrial mitral annular diastolic velocity (a') [cm/s]	9.82 ± 2.28	10.21 ± 2.18	9.62 ± 2.31	0.088
E/e' lateral ratio	8.51 ± 2.63	9.10 ± 2.89	7.94 ± 2.71	0.025
Diastolic dysfunction grade I no. (%)	7 (3.5)	2 (2)	6 (6)	0.12
Diastolic dysfunction grade II no. (%)	102 (51)	47 (49)	55 (53)	0.31
Diastolic dysfunction grade III no. (%)	0	0	0	-
Right ventricular structure and function				
Right atrial volume single plane [ml/m^2^]	23.7 ± 9.0	22.4 ± 8.7	25.2 ± 9.2	0.033
Mid-RV diameter [cm]	3.9 ± 0.5	4.0 ± 0.5	3.9 ± 0.5	0.34
Tricuspid annular plane systolic excursion ^[cm]	2.65 ± 0.45	2.62 ± 0.43	2.67 ± 0.45	0.47
Peak tricuspid regurgitation gradient ~ [mmHg]	18.2 ± 7.2	17.4 ± 6.8	18.5 ± 7.3	0.44

Our plasma NT-proBNP cut-off value has previously been defined but represented the median P-NT-proBNP in the first 50 patients examined in the study [10].

p-values reflect comparison between patients with high and low plasma NT-proBNP

Data are expressed as means ± SD or medians, interquartile range*

Left ventricular (LV)

Right ventricular (RV)

^no patients had tricuspid annular plane systolic excursion less than 1.5 cm

~ 65 of all patients had no tricuspid regurgitation, 111 had discrete and 5 patients had mild tricuspid regurgitation. In 19 patients we were not able to evaluate tricuspid regurgitation.

### LV structure and function

LV mass, LV hypertrophy indexed to BSA/height and height^2.7 ^and LV geometry are shown in Table 3. Moderate-severe LV hypertrophy was more common when indexed to ideal body size, e.g.: LV hypertrophy according to LV mass/height^2.7 ^criteria was present in 24% (48/200) of patients, including 9% (18/200) of patients with moderate-severe LV hypertrophy. Whereas LV hypertrophy according to LV mass/BSA criteria was only seen in 10% of patients, including 4% (7/200) of patients with moderate-severe LV hypertrophy. In addition, normal LV geometry was present in 36% of patients and the distribution of concentric hypertrophy, eccentric hypertrophy and concentric remodelling were 10%, 1% and 52% of patients, respectively. Only 3% (5/200) had moderate-severe enlarged diastolic LV volume (not shown).

**Table 3:** Left ventricular mass, hypertrophy as defined by indexing of body surface area, regular height and height^2.7^, and geometry in 200 type 2 diabetic patients with elevated urinary albumin excretion rate

Left ventricular (LV) hypertrophy in women (n = 46)*	Normal no. (%)	Mild no. (%)	Moderate no. (%)	Severe no. (%)
LV mass, g, 67-162, 163-186, 187-210 and ≥ 211	24 (52)	10 (22)	7 (15)	5 (11)
LV mass/BSA, g/m^2^, 43-95, 96-108, 109-121 and ≥ 122	40 (87)	3(7)	1(2)	2 (4)
LV mass/height, g/m, 41-99, 100-115, 116-128 and ≥ 129	24 (52)	14 (30)	5 (11)	3 (7)
LV mass/height^2,7^, g/m^2,7^, 18-44, 45-51, 52-58 and ≥ 59	28 (61)	14 (30)	2 (4)	2 (4)
Relative wall thickness, cm, 0.22-0.42, 0.43-0.47, 0.48-0.52 and ≥ 0.53	19 (41)	8 (17)	9 (20)	10 (22)
LV hypertrophy in men (n = 149)*	Normal no. (%)	Mild no. (%)	Moderate no. (%)	Severe no. (%)
LV mass, g, 88-224, 225-258, 259-292 and ≥ 293	111 (75)	21 (14)	8 (5)	9 (6)
LV mass/BSA, g/m^2 ^49-115, 116-131, 132-148 and ≥ 149	135 (91)	10 (7)	2 (1)	2 (1)
LV mass/height, g/m, 52-126, 127-144, 145-162 and ≥ 163	116 (78)	17 (11)	11 (7)	5 (3)
LV mass/height^2,7^, g/m^2,7^, 20-48, 49-55, 56-63 and ≥ 64	119 (80)	16 (11)	10 (7)	4 (3)
Relative wall thickness, cm, 0.24-0.42, 0.43-0.46, 0.47-0.51 and ≥ 0.52	56 (38)	27 (18)	29 (19)	37 (25)

LV mass was calculated using the formula = 0.8 1.04[(LVIDd + PWTd + SWTd)3 - (LVIDd)3] + 0.6 g and indexed to body surface area (BSA), regular height and height^2.7^. LV relative wall thickness; 2 × PWd/LVDd. Reference limits are shown in the table.

* of the 200 patients, we did not perform echocardiography in two men and in addition it was no possible to measure left ventricular mass in two women and one man due to poor echocardiographic windows

Five% (10/200) of patients had LV systolic dysfunction. In contrast, LV diastolic dysfunction was detected in 55% (109/200) patients while 37 had normal diastolic echocardiographic findings and 54 were categorized as displaying indeterminate diastolic dysfunction according to presence of only 1 or 2 tissue Doppler criteria for LV diastolic dysfunction (n = 42) or because of incomplete diastolic measurements (n = 12). Among the 109 patients who had LV diastolic dysfunction, impaired LV relaxation (grade I diastolic dysfunction) was detected in 4% (7/200) and a pseudonormal LV relaxation pattern (grade II) was detected in 51% (102/200) of subjects. Restrictive physiology (grade III) was not noted in any patient. Finally, LV E/e'ratio was increased ( > 10) in 24% (48/200) of patients, and these patients did not have increased LA volumes compared to patients with normal E/e'ratio. Associations between CV risk factors and echocardiographic abnormalities were mostly weak and not significant after adjustment for age and sex (not shown). However, we demonstrated a strong independent correlation between LV diastolic dysfunction and poor glycaemic control (the adjusted OR of having LV diastolic dysfunction was 2.42 (1.36-4.31) per 1% increase of HbA_1c _after adjustments for age, sex and all described CV risk factors, p = 0.003).

### Atrial size

LA volume was abnormal in 27% of (54/200) patients, with moderate-severe LA dilatation in 7% (14/200) of patients, where as RA volume was abnormal in 23% (45/200) of patients, including 9% (18/200) of subjects with moderate-severe RA dilatation.

### Plasma NT-proBNP levels and echocardiographic abnormalities

Echocardiographic variables in patients with P-NT-proBNP levels below and above cut-off value are described in Table 1. Subjects with elevated P-NT-proBNP levels did not have more LV hypertrophy, manifest atrial dilatation and LV dysfunction than patients with NT-proBNP levels below this value (p > 0.05). We did, however demonstrate that patients with P-NT-proBNP levels above cut-off value had higher LA volume index within the normal limits (26.7 ± 6.7 vs. 24.5 ± 5.8 ml/m^2^, p = 0.021), higher RA volume index (25.2 ± 9.2 vs. 22.4 ± 8.7 ml/m^2^, p = 0.033) and increased E/e'ratio (9.10 ± 2.89 vs. 7.94 ± 2.71, p = 0.003) than patients with NT-proBNP levels below this value. LA or RA volume index remained significantly different after adjustment for age, sex and CV risk factors shown in Table 2 but not when significant CAD was introduced (p > 0.05). We also demonstrated a borderline lower peak systolic performance (8.28 ± 1.54 vs. 8.71 ± 1.60 cm/s, p = 0.057) in patients with P-NT-proBNP levels above our cut-off value. LV mass indexed to height^2.7 ^was not significantly different among the two groups (p > 0.05).

### Associations between echocardiographic variables and significant CAD

In total, 70 high risk patients had significant CAD defined by abnormal MPI and/or CAG, representing 35% (70/200) the population.

The capabilities of selected echocardiographic variables to predict significant CAD were assessed in the two logistic regressions models and expressed as adjusted ORs. In both models elevated LV mass/height^2.7 ^even within normal values was the only independent predictor of significant CAD, (adjusted ORs were 1.88 [1.12-3.15] and 2.50 [1.19-5.23] per 10 g increase for model 1 and 2, respectively). Importantly, the strong independent association between LV mass/height^2.7 ^and significant CAD was also seen in both models without inclusion of the low risk patients (p = 0.015). Finally, patients with LV hypertrophy (as defined by LV mass/height^2.7^) had an OR of 4.53 (1.14-18.06) for significant CAD (model 1).

## Discussion

### Principal findings

We determined the prevalence of echocardiographic abnormalities, including LV hypertrophy, RA and/or LA dilatation, and LV systolic and diastolic dysfunction, respectively, and their relation to P-NT-proBNP levels or significant CAD in asymptomatic type 2 diabetic patients with microalbuminuria that received intensive multifactorial treatment. In these patients, P-NT-proBNP levels were not associated with echocardiograpic abnormalities. LV hypertrophy was present in 24% when indexed to height^2.7 ^and LV diastolic dysfunction was observed in 55% of patients. Finally, LV mass index to height^2.7 ^and LV hypertrophy but not LV diastolic dysfunction were strongly and independently associated with significant CAD.

### Plasma NT-proBNP and echocardiographic abnormalities

We have previously demonstrated a strong and independent risk for CV mortality with elevated P-NT-proBNP in type 2 diabetic patients [3], and in the present study we therefore investigated if echocardiographic abnormalities, that holds important prognostic value, were more common in patients with increased P-NT-proBNP levels [5]. Our subjects with increased P-NT-proBNP levels did not have more LV hypertrophy, manifest atrial dilatation and LV systolic or diastolic dysfunction but we demonstrated that left and right atrial volume indexes within the normal limits were independently higher in patients with increased NT-proBNP. In animal models, BNP gene expression reflect atrial and ventricular pressures [22] In elderly human subjects LA > 32 ml/m^2 ^was associated with an increased risk of heart failure [23]. However, this cut-off represents 2 SDs from the normal mean LA volume of 22 ± 5 ml/m^2 ^and it is therefore difficult to translate our of finding of 2.2 ml/m^2 ^difference between the two groups. In contrast, the LA dilates in response to the chronic increased LV and LA filling pressures and therefore as such is a sensitive cumulative marker of diastolic dysfunction. Accordingly, a small structural change of LA volume could therefore reflect long-term risk (NT-proBNP) rather than the functional diastolic echocardiographic parameters, which are more subject to high fluctuation with preload and other short term conditions. This could also explain why NT-proBNP was not related to diastolic dysfunction as described in the present study. This is in line with some studies [24], however other studies have shown that BNP is an early determinant of both diastolic and systolic dysfunction. Specifically, in asymptomatic newly diagnosed diabetic patients, it has been demonstrated that BNP is associated with the presence of diastolic dysfunction [4]. Accordingly, the use of BNP in the screening for preclinical moderate-severe diastolic dysfunction in diabetic patients has been suggested [25]. Finally, BNP is increased in subjects with systolic dysfunction in the general population [26]. The prognostic value of right atrial volume index is even more scarcely described but the right atrial association with NT-proBNP has previously been reported in other subjects [27]. The association in the latter study, was particular important when pulmonary hypertension occurred as a result of chronic obstructive pulmonary disease [27]. Therefore a pulmonal component of poor prognosis might be revealed in this study, however the true meaning of NT-proBNP, left and right atrial volumes will be examined in our planned follow-up of these patients. Other limitations may explain why patients with increased NT-proBNP levels did not have more major echocardiographic abnormalities in the present study. First, patients in the present study were without prior heart disease and all patients had normal P-creatinine where as subjects in our earlier study with higher prevalence of echocardiographic abnormalities included all type 2 diabetic patients followed in an out-patient clinic (4). Second, the increasingly intensive treatment used today in our patients is likely to have influenced the results compared to the earlier study [28], since almost all of our patients received statins, aspirin, and RAAS blockade. Noteworthy, in the Steno-2 study, the P-NT-proBNP levels increased over time in all patients, but was less in the intensive therapy group [28]. Furthermore, as shown in Table 2, some modifiable risk factors targeted by the multifactorial treatment were lower in patients with high NT-proBNP (cholesterol, bloodpressure but not haemoglobinA1c), where as the non-modifiable risk factors such as diabetes duration, plasma creatinine and signs of peripheral artery disease were higher in patients with high NT-proBNP. Along this line and as discussed below, the low prevalences of echocardiographic moderate-severe abnormalities in this study might also dilute the putative associations with NT-proBNP levels. Third, other factors such as cardiac autonomic neuropathy, is likely to influence CV mortality. Of note, we have previously reported the correlations between NT-proBNP and significant CAD and/or atherosclerosis in different territories. Along this line, NT-proBNP levels might be a more sensitive marker of early asymptomatic prognostic ventricular disease than the echocardiographic examination.

### Echocardiographic abnormalities and intensive multifactorial treatment

Individual and multifactorial intensive interventions including polypharmacologic therapy according to the Steno-2 study is the current standard of care in our institution and is known to reduce CV morbidity and mortality [1]. Noteworthy, an audit compared the levels of treatment targets before and after the Steno-2 study (2002 and 2009) and demonstrated that the Steno-2 study results of lowering haemoglobinA1c, lipids and blood pressure were successfully implemented into clinical practise at Steno Diabetes Center [29]. Almost all patients in the current study received statins, aspirin and RAAS blockading agents, and 33%, 28%, and 17% received 2, 3, and ≥ 4 antihypertensive drugs on top of RAAS blockade. Indeed, this intensive treatment yielded mean total cholesterol levels of 3.9 mM, arterial blood pressures of 130/75 mmHg, and haemoglobinA1c levels of 7.9%, respectively, which may explain why most of the conventional CV risk factors as well as levels of albuminuria were not associated with echocardiographic variables in our study (Table 2).

The reported prevalence of LV hypertrophy depends on classification criteria and population characteristics, ranging from 3% in the normotensive general population to almost 75% in hypertensive patients. The LV hypertrophy diagnosis varies with the methods, formulas, indexing and cut-off values that are applied. In a recent relatively large echocardiographic study with use of equivalent methods and classification schemes as used in our current study, moderate-severe LV hypertrophy (g/m^2^) was present in 13% of 305 type 2 diabetic patients [16]. That study included asymptomatic patients referred to a diabetes clinic for the first time with less than 5 years of diabetes duration and without prior CV disease. In comparison, moderate-severe LV hypertrophy (g/m^2^) was only seen in 4% of our cases despite that our patients had microalbuminuria and frequently demonstrated other signs of microvascular disease (retinopathy and neuropathy) with average diabetes duration of 13 years. In a previous study with use of different echocardiographic methods and cut-off values, we reported that LV hypertrophy (g/m^2^) was present in 75% or 51% of type 2 diabetic patients with or without diabetic nephropathy, respectively, and in 9% of controls [30]. In another study that included 426 normoalbuminuric type 2 diabetic patients without cardiovascular disease and not taking antihypertensive treatment, we also showed that LV hypertrophy (g/m^2.7^) was present in 43% of subjects [7]. Had these previously used criteria been used in the present study we would have demonstrated LV hypertrophy in 21% (g/m^2^) or 46% (g/m^2.7^) of patients, respectively (data not shown). The similar prevalence of LV hypertrophy in patients with normoalbuminuria as observed in our previous study (5) and the higher prevalence of LV hypertrophy in newly referred diabetic patients [16], compared to our complicated patients with microalbuminuria suggests that the prevalence of LV hypertrophy has been reduced by multifactorial treatment. This is in line with our recent finding that the prevalence of carotid intima-media thickness was indeed lower than expected in the present cohort [8]. The beneficial effect of antihypertensive agents on LV hypertrophy in diabetics is well-established, although the regression of LV hypertrophy in these patients appears to be less than in subjects without diabetes [31]. LV hypertrophy and even increased LV mass within normal values were associated with significant CAD, and this observation may contribute to the previously described higher CV mortality in diabetic patients with residual LV hypertrophy despite treatment [9,32]. Whether additional treatment aimed at reduction of LV hypertrophy in diabetes patients on intensive multifactorial treatment (with or without CAD) can ameliorate prognosis remains to be determined.

Our finding of more than 50% of patients with LV diastolic dysfunction in the present cohort was not unexpected, since LV diastolic dysfunction is considered to be resistant to medical treatment and prevalent in diabetic patients [5,16]. The pathogenesis of LV diastolic function is not known in detail, but a contribution of increased afterload, e.g. due to augmented arterial stiffness and microvascular disease, reduced myocardial perfusion, and increased myocardial stiffness associated with extracellular matrix alterations, fibrosis and metabolic derangements have been suggested [33]. Interestingly, LV diastolic dysfunction was not related to LV mass or carotid compliance in patients with or without significant CAD, but a strong association with glycaemic control (HbA1c levels) was found. This finding is in accordance with the hypothesis that hyperglycaemia can induce increased myocardial stiffness, e.g. by extracellular advanced glycation products leading to myocardial fibrosis [33]. No patients had restrictive LV diastolic dysfunction and only 4 patients had E/e' > 15 which would indicate severely increased LV filling pressures and is considered to be pathognomonic for diastolic heart failure in the presence of heart failure symptoms and normal LV systolic function [27]. The prevalence of moderate-severe LA dilatation was also low and particular severely abnormal LA volume ( ≥ 40 ml/m^2^) was only observed in 5 patients. A recent study reported LA distension ( > 32 ml/m^2^) in almost one third of patients with a lower CV risk profile compared to our study cohort [16]. These observations add to the notion that intensive multifactorial treatment may reduce the detrimental cardiac consequences of diabetes and hereby potentially delay clinical heart failure and reduce CV mortality. However, as stated above the true clinical impact of the presence of echocardiographic abnormalities in our present cohort of aggressively treated patients will be examined by planned prospective follow-up.

## Conclusion

Among asymptomatic type 2 diabetic patients with microalbuminuria that received intensive multifactorial treatment, patients with increased P-NT-proBNP levels did not have more echocardiographic abnormalities. LV diastolic dysfunction was frequently observed, where as LV hypertrophy was less frequent and associated with significant CAD.

## Abbreviations

ABI: Ankle-brachial index; ACE: Angiotensin-converting enzyme; BMI: Body mass index; BSA: Body surface area; CAD: Coronary artery disease; CAG: Coronary angiography; CCS: Coronary calcium score; CI: Confidence interval; CIMT: Carotid intima-media thickness; CTA: CT angiography; CV: Cardiovascular; DT: Deceleration time; IVRT: Isovolumetric relaxation time; LVEF: Left ventricular ejection fraction; LVIDd: Left ventricular inner diameter in diastole; MPI: Myocardial perfusion imaging; NT-proBNP: N-terminal pro brain natriuretic peptide; OR: Odds ratio; PWTd: Posterior wall thickness in diastole; RAAS: Renin-angiotensin-aldosterone-system; RHI: Reactive hyperaemia index; ROC: Receiver operating characteristic; SD: Standard deviation; SWTd: Septal wall thickness in diastole; TBI: Toe-brachial index.

## Authors' contributions

HR: researched data, contributed to discussion, wrote manuscript. PKJ, PR, PRH, H-HP, NW, AK, CLP, KW: researched data, contributed to discussion, reviewed/edited manuscript. All authors read and approved the final manuscript.

## Sources of funding

The study was kindly supported by The European Foundation of the Study of Diabetes (EFSD).

## Disclosures

Competing interests: Dr. Rossing reports having received lecture fees from Novartis and Boehringer Ingelheim, and research grant from Novartis, has served as a consultant for Merck, and having equity interest in NovoNordisk. Dr. Parving reports having served as a consultant for Novartis, Merck, Pfizer and Sanofi-Aventis, having equity interest in Merck and NovoNordisk and having received lecture fees from Novartis, Merck, Pfizer and Sanofi-Aventis. Dr. Parving has received grant support from Novartis, AstraZeneca and Sanofi-Aventis.
